# Novel microsatellite markers suggest significant genetic isolation in the Eastern Pacific sponge *Aplysina gerardogreeni*

**DOI:** 10.1007/s11033-023-09043-7

**Published:** 2024-01-06

**Authors:** Manuel Ricardo Salas-Castañeda, Nancy C. Saavedra-Sotelo, José Antonio Cruz-Barraza, Celia Isabel Bisbal-Pardo, Axayácatl Rocha-Olivares

**Affiliations:** 1 Posgrado en Ciencias del Mar y Limnología, Universidad Nacional Autónoma de México, Av. Universidad 3000, Ciudad Universitaria Coyoacán, C.P. 04510 Mexico City, Mexico; 2 Unidad Académica Mazatlán, Instituto Ciencias del Mar y Limnología, Universidad Nacional Autónoma de México, Av. Joel Montes Camarena s/n, CP 82000 Mazatlán, Sinaloa Mexico; 3 Facultad de Ciencias del Mar, Universidad Autónoma de Sinaloa (UAS), Mazatlán, Sinaloa Mexico; 4 Departamento de Oceanografía Biológica, Centro de Investigación Científica y de Educación Superior de Ensenada (CICESE), Ensenada, Baja California Mexico

**Keywords:** Porifera, Verongimorpha, Genetic connectivity, Primer development, Molecular markers

## Abstract

**Background:**

The Eastern Tropical Pacific (ETP) harbors a great diversity of Porifera. In particular, the *Aplysina* genus has acquired biotechnological and pharmacological importance. Nevertheless, the ecological aspects of their species and populations have been poorly studied. *Aplysina gerardogreeni* is the most conspicuous verongid sponge from the ETP, where it is usually found on rocky-coralline ecosystems. We evaluated the polymorphism levels of 18 microsatellites obtained from next-generation sequencing technologies. Furthermore, we tested the null hypothesis of panmixia in *A. gerardogreeni* population from two Mexican-Pacific localities.

**Methods and results:**

A total of 6,128,000 paired reads were processed of which primer sets of 18 microsatellites were designed. The loci were tested in 64 specimens from Mazatlan, Sinaloa (N = 32) and Isabel Island, Nayarit (N = 32). The microsatellites developed were moderately polymorphic with a range of alleles between 2 and 11, and *Ho* between 0.069 and 0.785. Fifteen loci displayed significant deviation from the Hardy–Weinberg equilibrium. No linkage disequilibrium was detected. A strong genetic structure was confirmed between localities using hierarchical Bayesian analyses, principal coordinates analyses, and fixation indices (*F*_*ST*_ = 0.108*). All the samples were assigned to their locality; however, there was a small sign of mixing between localities.

**Conclusions:**

Despite the moderate values of diversity in microsatellites, they showed a strong signal of genetic structure between populations. We suggest that these molecular markers can be a relevant tool to evaluate all populations across the ETP. In addition, 17 of these microsatellites were successfully amplified in the species *A. fistularis* and *A. lacunosa*, meaning they could also be applied in congeneric sponges from the Caribbean Sea. The use of these molecular markers in population genetic studies will allow assessment of the connectivity patterns in species of the *Aplysina* genus.

**Supplementary Information:**

The online version contains supplementary material available at 10.1007/s11033-023-09043-7.

##  Introduction

Sponges (Phylum Porifera) have become an interesting model for studying ecological and evolutionary processes in marine environments; they are mainly characterized by limited larval dispersal capacity, sexual and asexual reproduction, and a sessile lifestyle [1–5]. Due to their high abundance and wide diversity, they have a pivotal ecological role in most aquatic ecosystems, filtering the water column and providing substrate and shelter for a wide variety of organisms [3, 6, 7]. Despite its relevance, biological aspects such as reproduction and population genetics have generally been little studied e.g., [8–12].

Studies of the population genetics in sponge species have been conducted mainly on traditional nuclear and mitochondrial genes (e.g.: ITS’s, 28 S, 18 S, COI, among others), which showed a low polymorphism level in most groups. Therefore, the approaches addressed with these markers have been mostly in taxonomy and systematics, limiting the knowledge of historical and contemporary demography, as well as the phylogeographic patterns in this group e.g., [13, 14]. Nevertheless, the use of Next Generation Sequencing (NGS) platforms in the development of hypervariable markers, such as microsatellites, has increased significantly in population genetic studies of Porifera e.g., [15, 16]. These markers have shown to be powerful tools for population studies providing valuable information about their population dynamics e.g., [9, 11, 12].

Genetic population studies on Porifera have evaluated the degree of structure and connectivity genetic among populations. These patterns have allowed to identification of marine areas that function as genetic reservoirs [10]. The identification of these reservoirs provides fundamental information for designing management plans and protection of marine areas [10]. In addition, the patterns of structure and connectivity genetic have been used to estimate and evaluate the invasive potential of some sponge species [17], as well as the effects of mass mortality [9], the effects of hydrodynamics on the distribution of populations e.g., [18, 19], and the assessment of endangered species [20].

The genus *Aplysina* is the most conspicuous sponge group of the order Verongiida, with 47 valid species [21], many of which are widely recognized for their developed natural metabolites with cytotoxic and antimicrobial activity, and the use in bioengineering in regeneration tissue [22–26]. Despite its importance, some biological and ecological characteristics of this group of species are poorly understood. Although evolutionary aspects have been studied through mitochondrial and nuclear markers, they have exhibited low levels of polymorphism, even at the mitogenome level, which has limited their use in intraspecific studies [13, 27]. Species of this genus are characterized by larvae dispersal. The type of larvae has been described as clavablastula ciliated and swimming, with a period of settlement in the substrate after seven days [28]. Furthermore, this group presents asexual reproduction, which is carried out through fragmentation; where a part of the body of the sponge is detached and transported away several meters from the original parental site, where they settled and developed [29, 30]. These features allow hypothesized a low dispersal potential in *Aplysina* species.

The Eastern Tropical Pacific (ETP) is a region that extends from the Gulf of California to northern Peru, characterized by marine currents that provide unique oceanographic conditions promoting high levels of productivity and biodiversity [31, 32]. *Aplysina gerardogreeni* (Gómez and Bakus, 1992) is the most common verongid species from the ETP, usually found in rocky and coralline ecosystems [13, 33, 34]. Due to its high prevalence, the present study aims to develop microsatellite-type molecular markers to evaluate the genetic pattern of *A. gerardogreeni* in the ETP.

## Materials and methods

### Next-generation sequencing and microsatellite design

The procedure details for the NGS and specimen collections were previously described [27]. For microsatellite design, repetitive motifs of di-, tri-, and tetranucleotides were searched in the assembled contigs for a subsequent primer design using Msatcommander software [35]. All forward primers included the M13 primer sequence attached to their 5’ end following a protocol of dye-labeled universal primer [36].

### Sample collection and DNA extraction

**Fig. 1:**
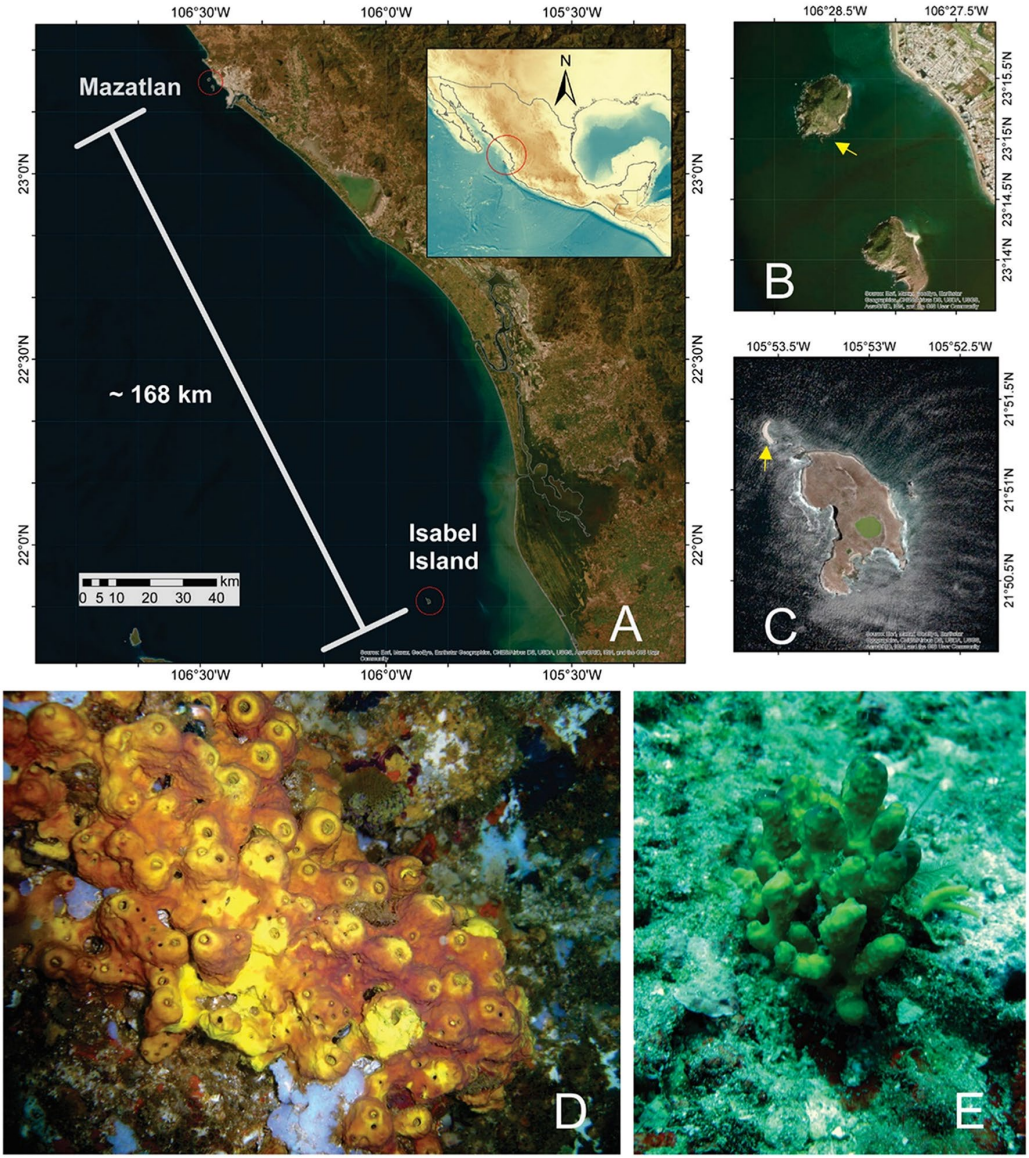
Location of the sampling stations in the Mexican Pacific and specimens’ images of *Aplysina gerardogreeni*. **A** Geographic distance between Mazatlan and Isabel Island. **B** Mazatlan. **C** Isabel Island. **D** *A. gerardogreeni* from Mazatlán. **E** *A. gerardogreeni* from Isabel Island (the yellow arrows indicate specimen collection sites for both sites). (Color figure online)

Sixty-four specimens of *A. gerardogreeni* were collected by SCUBA diving, 32 from Mazatlán Bay (23°15′29″N, 106°28′25″W) and 32 from Isabel Island (21°51′15″N, 105°53′33″W) Mexico (Fig. 1); samples were collected in different years (from 2010 to 2021) (Table S1). Genomic DNA was obtained using Promega’s Wizard® SV Genomic DNA Purification System protocol following the manufacturer’s instructions.

###  Amplification and genotyping of microsatellites

The PCR reaction mix contained 0.7 µl dNTPs (10mM) (Promega™), 2.0 µl 5x PCR Buffer (Promega™), 1.1 µl MgCl_2_ (25 mM) (Promega™), 0.5 µl unlabeled M13-tailed F-primer (10 mM), 0.5 µl R-Primer (10 mM), and 1.0 µl of fluorochrome-labeled F-primer (10 mM) (FAM, VIC, PET or NED), 1.0 µl BSA (20ng/µl) (Bovine Serum Albumin; SIGMA™), 0.1 µl Taq DNA polymerase (5u/µl) (Promega™), 1.5 µl of DNA (50 ng/µl), and fill out with H_2_O (styled Milli-Q Merck Millipore) to a final volume of 13 µl.

The thermocycling profile consisted of two stages: first 94 °C/4 min, followed by 30 cycles of 94 °C/30 s; 59 °C/30 s and 72 °C/60 s. Second, the fluorochrome-labeled forward primer was added to continue with 10 cycles of 94 °C/30 s; 53 °C/30 s and 72 °C/60 s; and a final elongation of 72 °C/20 min. Amplification products were visualized in 1.5% agarose gels stained with Gel Red™ Nucleic Acid Gel Stain (Biotium). To test the success of amplifying microsatellite loci on congeneric species, we randomly selected 3 specimens from each of the representative species of *A. lacunosa* and *A. fistularis* from the Mexican Caribbean and the Gulf of Mexico, respectively.

The final products were genotyped with an ABI3730 DNA analyzer (Applied Biosystems™). The genotypes were scored with Genemarker v3.0.1 software with GeneScan™ 500 LIZ (Soft Genetics, State College, PA, USA) and to convert and determine allelic size. The dataset to generate input-files was handled using a macro-Excel, Flexibin [37]. Finally, the presence of null alleles, large allele dropout and genotyping errors were assessed with Micro-Checker v.2.2.3 [38]. In addition, following the same methodology, we tested the cross-amplification in specimens of congeneric species: *A. fistularis* and *A. lacunosa*.

### Data analyses

Polymorphism levels were estimated by the observed (*H*_*O*_) and expected heterozygosity (*H*_*E*_) indices, the number of alleles per locus and the polymorphic content index (PIC) using a macro excel Mstools v 3 [39]. Further, we evaluated the linkage disequilibrium (LD) between pairs of loci using Mstools. Tests of Hardy–Weinberg Equilibrium (HWE) for each locus were assessed using a probability test with a level of significance determined by Markov chain parameters of 1,000 dememorization steps, 100 batches and 1,000 iterations per batch using GENEPOP Web v 4.2 [40], we used the Weir and Cockerham for *F-statistics* [41]. The *p*-values of multiple comparison analyses (HWE and LD) were adjusted using the classical one-stage method of the False Discovery Rate (FDR) procedure [42].

For the statistical analyses of genetic structure, we only used those microsatellite loci in HWE. First, we used Structure v 2.3.4 software [43] with parameters set to 10 iterations discarded as a burn-in, and 100,000 Markov Chains Monte Carlo (MCMC) were run with a burn-in of 10,000 iterations. Moreover, Structure was run using an admixture ancestral model with independence of allele frequencies, and prior information of sample location. Ten replicates were run for each *K* value (*K* = 1 to 2). The K number was estimated with Structure Harvester Web v 0.6.94 [44]. According to plots of log probability LnP(K) of the data (*Supplementary Material Fig. S1*), the ten replicates for the best K were merged in Clumpp [45] and visualized by Distruct [46]. Second, Principal Coordinates Analysis (PCoA) was constructed using a pairwise codominant genotypic distance matrix using GenAlEx v 6.5 [47]. Third, population differentiation was assessed using pairwise *F*_ST_ in GenAlEx v 6.5 [47].

## Results

### Genetic diversity

Forty-one microsatellite loci were isolated, of which 18 presented at least two alleles per locus (Table 1). Genetic diversity indices were performed within loci with at least three alleles in each location (Table 2). Large allele dropout and genotyping errors were not detected, but seven loci exhibited the presence of possible null alleles. Six loci were in HWE and neither locus showed linkage disequilibrium (Table 2).

**Table 1:** Summary statistics of microsatellite loci in *Aplysina gerardogreeni* from the Mexican Pacific

Locus (GenBank accession number)	Motif	Primer sequence (5′→3′)		Dye	Allelic range (bp)	*N*_*A*_
AGMX-194964 (OR553599)	(ACAT)4	F:	AGTATTGTTGTCCTTGGCCG	PET	122–170	9
		R:	TCTGTCAGAACACGTGCAC			
AGMX-191152 (OR553600)	(ACAT)4	F:	AAGAAACACACCTGCCCTAC	NED	102–170	10
		R:	TGGTGGTTGGTGTGGGAC			
AGMX-82588 (OR553601)	(ACAT)5	F:	ACACGGCATACCTACATACTC	FAM	160–180	5
		R:	TATCCGAACATGCTGACCAG			
AGMX-15843 (OR553602)	(AC)11	F:	TCTACATGCCAGACTAACAGC	VIC	106–122	9
		R:	TGGTTAAGTGCATGCATTTGTG			
AGMX-44589 (OR553603)	(ACGT)4	F:	AGTGCTGAACCTACATTTCTG	NED	120–148	7
		R:	CTGAAGCTCTCCAGTACCTG			
AGMX-8089 (OR553612)	(ACC)6	F:	CATAGAGGAGGGCTGTACTG	PET	114–132	5
		R:	AAGTGCATGCTTCACTGGAG			
AGMX-57397 (OR553604)	(TG)9	F:	GTGCTGTTCTCCCACTTGTG	FAM	144–154	4
		R:	TGAGTTCAGCATGATTCACTGC			
AGMX-37595 (OR553605)	(AG)10	F:	ACAGGCTACTATCAGTCCTCTC	VIC	125–135	5
		R:	TTGACAAAGCAGAGTTTCAGC			
AGMX-180680 (OR553613)	(ACAT)4	F:	AACATGTTTGCTTGCATTGG	PET	128–132	2
		R:	TCGTTCTACTGTCAACTCTAGC			
AGMX-31049 (OR553614)	(ACAG)7	F:	ACCACAACAGCCTGTACATG	PET	170–182	4
		R:	GTCCCGCATTGTATTTCACC			
AGMX-57158 (OR553606)	(ACGC)4	F:	TTTCTGCAAAGCTGTGGTTG	FAM	116–132	5
		R:	AGGAGCACTGTAATGATGAC			
AGMX-182674 (OR553607)	(ACGT)5	F:	AGATGCTGCCTTGTATTCAAC	VIC	113–129	5
		R:	CAGTAGTTCAGGTGTGCATG			
AGMX-145552 (OR553615)	(ACGC)4	F:	ACTGCACACACCACTTCTAC	VIC	127–167	5
		R:	ATGTGATCTCTCCATGTGTG			
AGMX-6292 (OR553608)	(AC)10	F:	GGAGGGTACAACGAGAGGTC	PET	106–136	11
		R:	GCGCAGTGGTCACATCTG			
AGMX-734 (OR553609)	(TGTA)4	F:	TGACACAATCTATCCTATCTCC	NED	120–140	5
		R:	AACAGAGCAGTTCAGTGAGG			
AGMX-24882 (OR553616)	(ACAT)4	F:	CGACTTTCTTGCTAAGCTGTC	FAM	156–176	3
		R:	GAAGTACGTACCTTGTGAGC			
AGMX-123455 (OR553610)	(TGTA)4	F:	ATATGGCAATTGAGTGACTTAC	VIC	117–165	5
		R:	TCGTGCAATGTCAGTTTCTG			
AGMX-89450 (OR553611)	(AG)8	F:	CTTTCCAGTGTTCCGTGAGC	PET	119–149	6
		R:	AGTAGGATCCTCGTGAGTAGC			
Total or average						5.8

*N*_*A*_ number of alleles (N = 64; 32 from Mazatlan Bay and 32 from Isabel Island, Mexico)

**Table 2:** Summary statistics of microsatellite loci with at least 3 alleles per locus detected in each locality from the Mexican Pacific

Locus	Mazatlan Bay						Isabel Island					Global					
	*PIC*	*N*_*A*_	*H*_*o*_	*H*_*E*_	*P*_*HWE*_	*F*_*IS*_	*PIC*	*N*_*A*_	*H*_*o*_	*H*_*E*_	*P*_*HWE*_	*F*_*IS*_	*PIC*	*H*_*o*_	*H*_*E*_	*P*_*HWE*_	*F*_*IS*_
AGMX-194964	0.406	3	0.400	0.504	0.016	0.223	0.697	8°	0.346	0.737	*P <* 0.001*	0.541	0.760	0.375	0.795	*P <* 0.001*	0.530
AGMX-191152	0.443	5°	0.344	0.528	*P <* 0.001*	0.362	0.755	8°	0.433	0.786	*P <* 0.001*	0.461	0.725	0.387	0.766	*P <* 0.001*	0.497
AGMX-82588	0.397	3°	0.094	0.514	*P <* 0.001*	0.822	0.662	5°	0.381	0.711	*P <* 0.001*	0.483	0.600	0.208	0.670	*P <* 0.001*	0.692
AGMX-15843	0.571	5°	0.406	0.640	*P <* 0.001*	0.378	0.743	8°	0.593	0.775	0.016*	0.249	0.765	0.500	0.801	*P <* 0.001*	0.377
AGMX-44589	0.442	5°	0.233	0.476	*P <* 0.001*	0.522	0.678	5	0.843	0.728	0.002*	− 0.143	0.639	0.548	0.688	*P <* 0.001*	0.203
AGMX-57397	0.396	3	0.524	0.500	1.000	− 0.023	0.298	3°	0.125	0.322	*P <* 0.001*	0.625	0.380	0.311	0.443	0.001*	0.299
AGMX-37595	0.677	5	0.793	0.725	0.207	− 0.076	0.452	5	0.428	0.494	0.447	0.150	0.727	0.614	0.769	*P <* 0.001*	0.202
AGMX-57158	0.524	3°	0.208	0.603	*P <* 0.001*	0.666	0.636	4°	0.400	0.689	0.001*	0.436	0.630	0.306	0.685	*P <* 0.001*	0.556
AGMX-182674	0.403	3°	0.043	0.515	*P <* 0.001*	0.919	0.699	5°	0.291	0.743	*P <* 0.001*	0.620	0.735	0.170	0.779	*P <* 0.001*	0.783
AGMX-6292	0.515	9	0.407	0.534	0.082	0.255	0.168	5	0.181	0.171	1.000	− 0.037	0.380	0.306	0.394	0.094	0.225
AGMX-734	0.590	3	0.733	0.665	*P <* 0.001*	− 0.086	0.609	5	0.900	0.668	*P <* 0.001*	− 0.331	0.620	0.817	0.689	*P <* 0.001*	− 0.186
AGMX-123455	0.261	3°	0.161	0.297	0.016	0.470	0.493	4°	0.406	0.580	0.004*	0.314	0.411	0.286	0.487	*P <* 0.001*	0.415
AGMX-89450	0.241	4	0.280	0.253	1.000	− 0.087	0.252	4°	0.125	0.263	0.005*	0.540	0.255	0.204	0.264	0.025*	0.228
Total or average	0.452	4.1	0.356	0.520		0.334	0.549	5.3	0.420	0.590		0.300	0.587	0.387	0.633		0.370

Mazatlan Bay (N = 32), Isabel Island (N = 32) and Global (N = 64) from the two study localities)

*N*_*A*_ number of alleles, *H*_*O*_ Observed heterozygosity index, *H*_*E*_ expected heterozygosity index, *P*_*HWE*_ *P* value of the Hardy-Weinberg equilibrium test, *F*_*IS*_ Inbreeding index, *PIC* polymorphic information content

°the presence of null alleles, *significant results after FDR-value adjustment (*P*-value < 0.05)

Almost all loci presented low levels of genetic diversity. The highest and the lowest numbers of alleles per locus were detected in the AGMX-6292 (11 alleles) and AGMX-180680 (2 alleles) loci. The PIC average in the data set was 0.587; the values for each locus were between low and moderate (0.255–0.765). The highest values of *H*_*O*_ were detected in AGMX-37595 and AGMX-734 (0.614 and 0.817) loci. The *F*_*IS*_ showed high values in loci AGMX-82588 (0.692) and AGMX-182674 (0.783), and AGMX-734 loci with exogamy (− 0.186; Table 2). Isabel Island presented higher values of genetic diversity than Mazatlan, except the *F*_*IS*_ average was higher in Mazatlan.

### Genetic differentiation

**Fig. 2:**
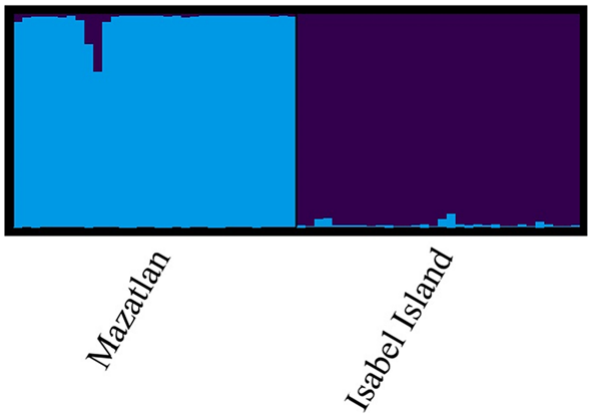
Probability of membership into genetic clusters (K = 2) by each sample and locality. Each vertical line represents a sponge with a probability of membership to each cluster (blue and purple). (Color figure online)

**Fig. 3:**
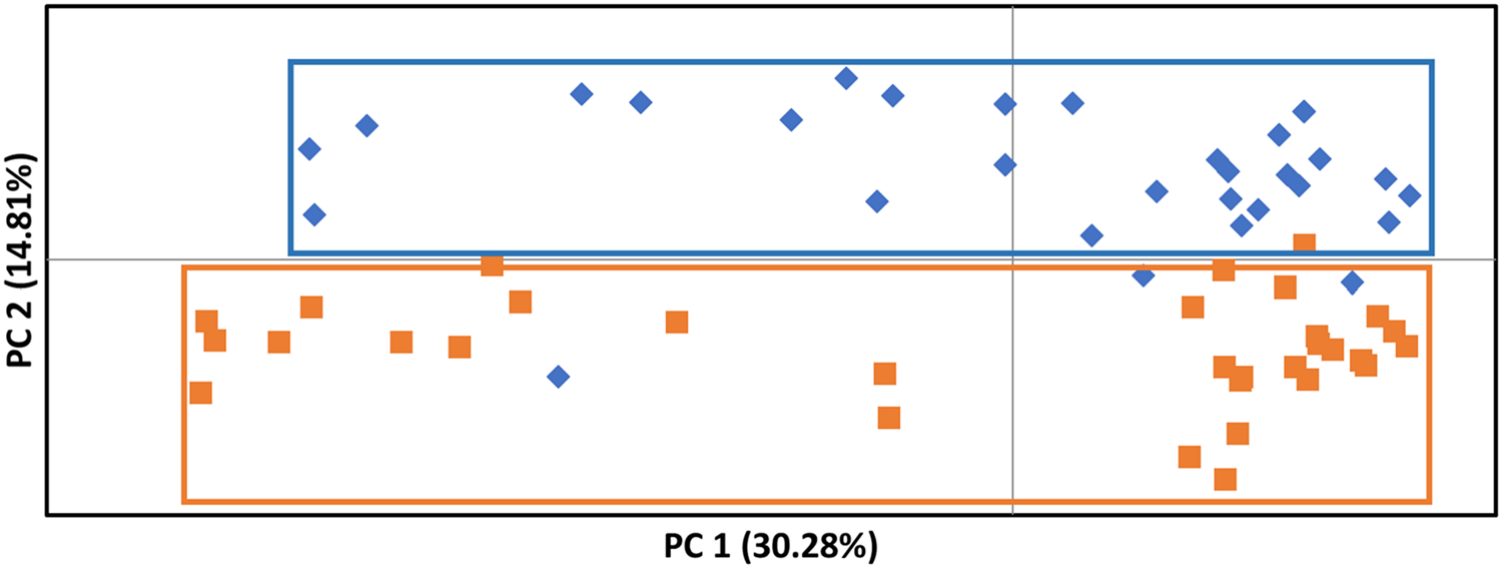
Principal coordinate analyses (PCoA) based on the genetic distance of sponges from Mazatlan Bay (blue diamonds) and Isabel Island (orange square). (Color figure online)

 The hierarchical Bayesian analysis revealed two genetic clusters among the organisms. The average LnP(K) value was maximal at 2, and the membership probabilities of the sample individuals reflected a clear geographical pattern of genetic differentiation (Fig. 2). In addition, the genetic distribution of the individuals was graphically represented under a vector plane in the PCoA, the results suggested one genetic group associated with each locality (Fig. 3). Nonetheless, there is an overlap of some Mazatlan individuals with Isabel Island cluster (Fig. 3). The initial two principal components (PCs) explain 45.09% of the observed variation: PC 1 explained 30.28%, whereas PC 2 explained 14.81%. The AMOVA showed a moderate genetic structure between localities (*F*_*ST*_ = 0.108; *P-value* < 0.05).

## Discussion

### Genetic diversity

The high number of loci out of HWE could be indicative of either technical issues, such as null alleles [48, 49] (Table S2), or biological features of this species, including the potential of inbreeding [28]. Although there is little information about the reproductive biology of *A. gerardogreeni*, one record in Isabel Island showed that 5.2% and 2.5% of samples developed oocytes and spermatic cysts, with a female-male sex proportion of 3:1 [50]. Those results indicate sexual reproduction in a small portion, leaving the possibility that most sponges of this species reproduce asexually. This would be consistent with low levels of genetic diversity and high values of inbreeding in both locations (Table 2).

In general, sponge species tend to present low levels of genetic diversity, which could be associated with the asexual reproduction seen in many species e.g., [51–53]. This type of reproduction is associated to the response of massive population reductions by meteorological phenomena such as storms and hurricanes, and hydrodynamic local events [8, 18, 19, 54]. Mazatlan and Isabel Island are localized in the mouth of Gulf of California, a region with high oceanographic dynamics, because converged oceanic currents such as the California Current near-surface and the Mexican Coastal Current at sub-surface [55]. In addition, this region is characterized by high activity of tropical cyclones and hurricanes [56]; therefore, it is probable that sponge species recover their populations after a drastic decline through asexual reproduction. Nevertheless, we did not detect identical genotypes across both localities. This finding could be due to (1) the sampling method possibly preventing collection of clones because there were from three to five meters of separation between samples, and (2) this species present both types of reproduction according to environmental conditions (stressful and non-stressful) [54]. To corroborate our findings, studies at smaller geographic scales must be conducted e.g., [12, 53].

### Genetic differentiation

Sponges from Mazatlan and Isabel Island conform to two genetically isolated populations (*F*_*ST*_ = 0.108*; Figs. 2 and 3). Although there is little evidence of sexual reproduction in *A. gerardogreeni*, it is possible exchange organisms between populations through larval dispersal by currents or by hitchhiking invasive and floating buds [8, 11, 12, 54]. Under the premise that *A. gerardogreeni* could develop a type of clavablastula larva like its congeneric (*A. aerophoba*) [28], it is possible there is limited dispersal. In addition, the environmental conditions between locations play a relevant role in fixing different alleles [57, 58]. Both localities present contrasting environmental conditions. Mazatlan is a coastal region near many estuaries and river mouths where sediment entrainment is characteristic; sponge species are suspension feeders and changes in sediment levels can affect the abundance of populations [59]. In contrast, Isabel Island present low sediment deposition and is not affected by anthropogenic impacts, thus is considered a site with better environmental conditions for reef communities [60].

To conclude, we observed a high degree of genetic structure in *A. gerardogreeni* using only six microsatellite loci; we hope to increase the number of microsatellites by increasing the study area at the Mexican Pacific (investigation in progress). In addition, these markers can be amplified in two congeneric species from the Caribbean Sea (*A. fistularis* and *A. lacunosa*), therefore, it offers the possibility of evaluating the patterns of genetic structure population in these species.

## Supplementary Information

Below is the link to the electronic supplementary material.

Supplementary material 1 (XLSX 11 kb)

## Data Availability

Information on the designated primers and microsatellite sequences will be available in GenBank once the manuscript is accepted.

## Declarations

### Research involving human and animal participants

No other studies with other animals or human participants were performed by any of the authors.
